# Whole-Body SPECT/CT versus Planar Bone Scan with Targeted SPECT/CT for Metastatic Workup

**DOI:** 10.1155/2017/7039406

**Published:** 2017-07-24

**Authors:** Olivier Rager, René Nkoulou, Nadia Exquis, Valentina Garibotto, Claire Tabouret-Viaud, Habib Zaidi, Gaël Amzalag, Stephanie Anne Lee-Felker, Thomas Zilli, Osman Ratib

**Affiliations:** ^1^Division of Nuclear Medicine, Geneva University Hospitals and Faculty of Medicine, University of Geneva, rue Gabrielle-Perret-Gentil, No. 4, 1211 Geneva, Switzerland; ^2^IMGE (Imagerie Moléculaire Genève), 20 chemin Beau Soleil, 1206 Geneva, Switzerland; ^3^Department of Radiology, University of California, Los Angeles, 200 Medical Plaza, Room 165-53, Los Angeles, CA 90095, USA; ^4^Division of Radiation Oncology, University of Geneva and Geneva University Hospitals, rue Gabrielle-Perret-Gentil, No. 4, 1211 Geneva, Switzerland

## Abstract

**Purpose:**

The use of SPECT/CT in bone scans has been widespread in recent years, but there are no specific guidelines concerning the optimal acquisition protocol. Two strategies have been proposed: targeted SPECT/CT for equivocal lesions detected on planar images or systematic whole-body SPECT/CT. Our aim was to compare the diagnostic accuracy of the two approaches.

**Methods:**

212 consecutive patients with a history of cancer were referred for bone scans to detect bone metastases. Two experienced readers randomly evaluated for each patient either planar images with one-field SPECT/CT targeted on equivocal focal uptakes (targeted SPECT/CT) or a whole-body (two-field) SPECT/CT acquisition from the base of the skull to the proximal femurs (whole-body SPECT/CT). The exams were categorized as “nonmetastatic,” “equivocal,” or “metastatic” on both protocols. The presence or absence of any extra-axial skeletal lesions was also assessed. The sensitivity and specificity of both strategies were measured using the results of subsequent imaging follow-up as the reference standard.

**Results:**

Whole-body SPECT/CT had a significantly higher sensitivity than targeted SPECT/CT to detect bone metastases (*p* = 0.0297) and to detect extra-axial metastases (*p* = 0.0266). There was no significant difference in specificity among the two approaches.

**Conclusion:**

Whole-body SPECT/CT is the optimal modality of choice for metastatic workup, including detection of extra-axial lesions, with improved sensitivity and similar specificity compared to targeted SPECT/CT.

## 1. Introduction

Bone scintigraphy remains a widely used imaging modality in the metastatic workup in patients with cancer, especially prostate, breast, and lung cancers. It is relatively inexpensive, allows whole-body screening, and is highly sensitive in the detection of malignant bone lesions. However, its specificity is limited by uptake in benign conditions (e.g., degenerative joint diseases, fractures, infections, and benign bone tumors) [1]. SPECT/CT (single-photon emission computed tomography/computerized tomography) has gained a wide acceptance for bone scanning. Many studies have shown that SPECT/CT reduces the rate of equivocal lesions compared to planar bone scan due to better anatomic localization of lesions and higher lesion-to-background contrast, with increased diagnostic accuracy over SPECT alone or planar scintigraphy alone [2–6].

Total acquisition times can be considerably increased by the addition of one or two SPECT/CT fields to planar scintigraphy. This can be a problem in elderly patients who cannot tolerate the supine position for extended periods. Furthermore, this can considerably reduce the daily throughput on the SPECT/CT camera, which is particularly relevant in a busy nuclear medicine department. A time-effective alternative would be to omit the planar images in favour of a whole-body SPECT/CT covering from the base of the skull to the proximal femurs.

The aim of this study was to compare the sensitivity and specificity of planar scintigraphy with one-field SPECT/CT targeted on equivocal focal uptakes (targeted SPECT/CT) and of whole-body (two-field) SPECT/CT acquisition alone. Whole-body SPECT/CT does not cover the whole skeleton, but only the proximal portions of the limbs: for this reason, we also compared the diagnostic performance of both modalities in detecting metastatic spread to the extra-axial skeleton.

## 2. Material and Methods

### 2.1. Patients

From October 2010 to August 2011, 212 consecutive patients referred for a bone scan for metastatic workup in the Nuclear Medicine Department of the University Hospital of Geneva were included in the study. Among them, 83 patients (60.8%) were female and 129 were male (40.2%) (mean age: 66.8 years old, range: 16–90). All of them had a history of histologically proven cancer; prostate (70 patients, 33%) and breast (50 patients, 23.6%) cancers were the most frequent followed by malignancies of bladder (25 patients, 11.8%), liver (20 patients, 9.4%), lung (11 patients, 5.2%), kidney (7 patients, 3.3%), and colon (4 patients, 1.9%). 15 patients (7.1%) had others malignancies and ten (4.7%) had unknown primary cancer. Four patients (1.9%) had two simultaneous neoplasia forms. The design of the present study was retrospective. The findings from whole-body SPECT/CT were compared with the results of subsequent imaging follow-up as the reference standard including CT, MRI, PET, or a subsequent scintigraphy ± SPECT/CT, as well as autopsy reports in some cases.

### 2.2. Planar Scintigraphy Acquisition

The patients received an intravenous dose of 10 MBq/kg with a fixed minimum of 400 MBq and a fixed maximum of 1500 MBq of ^99m^Tc-hydroxymethylene diphosphonate (^99m^Tc-HMDP) (Technescan, Mallinckrodt, USA). The mean dose (±SD) was 732 ± 172 MBq. Patients were asked to void before starting the acquisition. The whole-body planar scintigraphy was performed 3 hours after injection in the anterior and posterior projections, with a speed of 15 cm/min, on a double-head gamma camera with a multislice spiral CT scanner installed within the same gantry (Symbia T6, Siemens Medical Solutions, Germany). Whole-body views were acquired on the 140 keV photopeak with a 15% symmetrical window using a 256 × 1024 matrix.

### 2.3. Whole-Body SPECT/CT Acquisition

Whole-body SPECT/CT was performed for every patient, whether or not there were suspicious lesions on planar bone scintigraphy. Patients were asked to void before starting the acquisition. Arms were elevated over the head, if tolerated. Two consecutive acquisitions were performed, allowing coverage of the skeleton from the base of the skull to the proximal femurs, with some slight variations due to patient height. Immediately after the SPECT, an unenhanced CT scan was acquired. Both SPECT and CT were performed during shallow breathing, with the patients lying comfortably in the supine position.

For SPECT acquisition, counts from the 15% energy windows at 140 keV were acquired into a 128 × 128 matrix. The axial field of view of the camera was 38.7 cm (75.4 cm for two SPECT with 2 cm overlap). Sixty-four 15-second projections were acquired over 360 degrees using a noncircular orbit (autocontouring) in the step-and-shoot mode. The camera heads were equipped with a high-resolution low-energy parallel-hole collimator. Immediately after the SPECT data was acquired, the raw data were reconstructed into transaxial, coronal, and sagittal slices using e.soft reconstruction software (Siemens Healthcare, Germany). Iterative reconstruction was performed using ordered-subsets expectation maximization with 8 iterations and 8 subsets (Flash3D™, Siemens Healthcare, Germany). Images were smoothed with a 3D spatial Gaussian filter (1 mm full width at half maximum).

The unenhanced CT was acquired in a 512 × 512 matrix, 110 kVp, 0.8 sec rotation time, pitch-0.5, 6 × 2 mm collimation. Because only bone structures required analysis, the tube current was reduced to 40 mAs with intensity modulation activated (CARE Dose 4D, Siemens Healthcare) to minimize radiation exposure. Image reconstruction using a high-resolution reconstruction algorithm with a sharp filter (B70 kernel) resulted in images with a slice thickness of 3 mm for a 2 mm reconstruction increment. CT-based attenuation correction was used. The total duration of the two-bed SPECT/CT was approximately 22 minutes.

The average CTDIvol ± SD for the whole-body SPECT/CT was 3.76 ± 0.74 mGy. Dosimetry was estimated to 5.5 mSv for CT using ImPACT CT Patients Dosimetry Calculator (version 1.0.4, Imaging Performance Assessment on Computed tomography, http://www.impactscan.org) and to 3.99 mSv for radiotracer for a 70 kg adult (0.0057 mSv/MBq) according to ICRP [7].

The fused SPECT and CT images were interpreted using a diagnostic quality workstation equipped with DICOM viewing software (Osirix MD, Pixmeo SARL, Switzerland).

### 2.4. Targeted SPECT/CT Reconstruction

One 38.7 cm length SPECT/CT centered on the desired region (as requested by the interpreting physician based on planar scintigraphy findings) was reconstructed by an independent person into transaxial, coronal, and sagittal slices using e.soft reconstruction software (Siemens Healthcare, Germany). The reconstruction parameters were the same as for whole-body SPECT/CT: iterative reconstruction was performed using ordered-subsets expectation maximization with 8 iterations and 8 subsets (Flash3D, Siemens Healthcare, Germany), and images were smoothed with a 3D spatial Gaussian filter (1 mm full width at half maximum).

### 2.5. Image Data Analysis

Planar bone scintigraphy with targeted SPECT/CT and whole-body SPECT/CT covering from the base of the skull to the proximal femurs were randomly and independently reviewed by two different nuclear medicine physicians with, respectively, 8 and 11 years of experience. One read the planar scintigraphy with targeted SPECT/CT and the other reader interpreted the whole-body SPECT/CT alone without planar acquisition. The only clinical information provided to the reader was the type of primary cancer. First, each reader recorded his degree of confidence on a patient analysis with a three-point scale:* A* patient with no lesion or only benign or probably benign lesion;* B* equivocal patient with a least one equivocal lesion without any malignant lesion;* C* patient with at least one malignant or probably malignant lesion. Finally, the reader of each modality also took note of the presence or absence of extra-axial skeletal lesions according on the three-point scale* (A*,* B*, and* C)*, partially visualized on whole-body SPECT/CT due to limited field of view as previously described.

A standard method for analysis was defined as follows: the fused SPECT/CT was analyzed by reviewing the entire exam in transaxial slices, as it is used for anatomic imaging (CT, MRI), with the ability to correlate abnormal lesions on additional orthogonal views. Maximal Intensity Projection (3D MIP) images of SPECT were also analyzed systematically. Attenuation correction (AC) SPECT was used by default for fusion imaging. Non-AC SPECT was available for reconstruction artifacts (such as due to dense material in the spine). A lesion was categorized as benign if it did not follow the physiological ^99m^Tc-HMDP uptake pattern but was not thought to represent a tumor site. These lesions showed uptake of low intensity or were located in anatomical regions or structures that could be associated with nontumoral ^99m^Tc-HMDP uptake. Lesions categorized as malignant did not follow the physiological ^99m^Tc-HMDP uptake pattern but had focal uptake corresponding to a suspicious metastatic site or pattern. If readers could not decide whether a lesion was benign or malignant on the basis of these criteria, the lesion was equivocal.

All readers completed training covering the following differential diagnoses: osteoarthritis, fractures/traumatic injuries, osteomyelitis, benign bone disease (Paget's, fibrous dysplasia, osteoid osteoma, osteochondroma, and osteoma of the skull base and sinuses), normal variants (sternal angle, peridental uptake, and frontal hyperostosis), artifacts (interstitial injection, intravascular retention, urinary contamination, and attenuation by metal devices), pitfalls (bone superscan, osteomalacia, and paraneoplastic hypertrophic osteoarthropathy), and uptake in the soft tissues (myositis ossificans, heterotopic calcifications, subcutaneous injection, and retention in the urinary tract).

### 2.6. Statistical Analysis

All data from whole-body SPECT/CT and planar bone scintigraphy with targeted SPECT/CT were compared and analyzed. Sensitivities and specificities of the two modalities were compared using an unconditional exact test of equality for two related binomial proportions under StatXact11 (CYTEL, USA) [8]. Confidence Intervals (CI) on difference were based on the standardized statistic and inverting two 1-sided tests under StatXact11 (CYTEL, USA) [9]. Receiver operating characteristic (ROC) analysis was performed using two thresholds: threshold* A* if equivocal result was considered positive for bone metastasis and threshold* B* if equivocal result was considered negative for bone metastasis.

## 3. Results

During the median follow-up period (±SD) of 17.5 ± 19.1 months (range: 4.5–56) of the 212 consecutive patients included in this study, bone metastases were confirmed in 43 patients (20%) and excluded in 169 patients (80%).

Based on planar with targeted SPECT/CT, 167 patients (78.8%) were negative for bone metastases, 5 (2.3%) were equivocal, and 40 (18.9%) were positive. On whole-body SPECT/CT, 163 (76.9%) patients were negative for bone metastases, 8 (3.8%) were equivocal, and 41 (19.3%) were positive. Results for metastatic status with whole-body SPECT/CT and planar with targeted SPECT/CT are presented in Table 1. The sensitivity was significantly higher with whole-body SPECT/CT than with targeted SPECT/CT, but there was no significant difference in specificity (Table 2). The analysis of the two most significant subgroups of patients with prostate cancer (*n* = 70) and breast cancer (*n* = 50) showed a higher sensitivity for whole-body SPECT/CT, but the comparison with targeted SPECT/CT did not reach significance due to smaller effectives (sensitivity of 100% versus 90.5%, *p* = 0.2 and 100% versus 78.6%, *p* = 0.1, resp.). Six patients were classified as “super bone scan” based on planar with targeted SPECT/CT, and all of them were concordant according to whole-body SPECT/CT.

Finally, among the 212 patients, 23 (10.8%) had at least one suspicious uptake in the extra-axial skeleton based on planar scintigraphy with targeted SPECT/CT, compared to 28 (13.2%) on whole-body SPECT/CT. Whole-body SPECT/CT status for extra-axial lesions and planar with targeted SPECT/CT results are presented in Table 3. All 23 patients having lesions classified as metastatic based on planar scintigraphy with targeted SPECT/CT also had proximal limb metastases identified on whole-body SPECT/CT. Four patients were classified as equivocal on planar scintigraphy with targeted SPECT/CT: 3 proximally located, all of which were also visible and classified as equivocal on the whole-body SPECT/CT. Only one lesion was not seen on SPECT/CT because it was outside the field of view, but the analysis of previous bone scans in the same patient showed that this lesion was stable over 28 months and was ultimately considered benign. Moreover, 6 patients (2.8%) without extra-axial metastasis on planar scintigraphy with targeted SPECT/CT were positive for extra-axial metastasis on whole-body SPECT/CT. The sensitivity was significantly higher with whole-body SPECT/CT than with targeted SPECT/CT to detect extra-axial metastases. There was no significant difference in specificity between the two imaging modalities (Table 4).

## 4. Discussion

**Figure 1:**
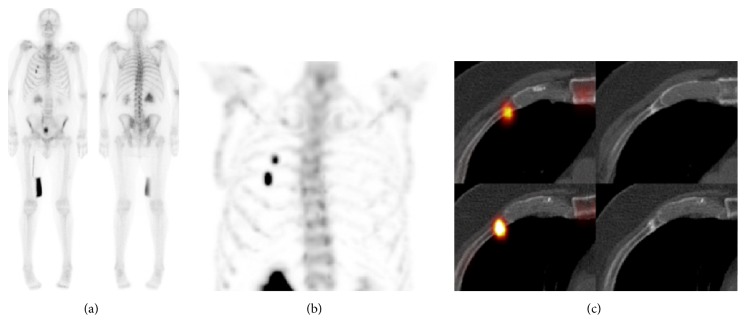
Metastatic workup for prostate cancer. Based on planar scintigraphy (a), a targeted SPECT/CT was acquired of the thorax. MIP of the SPECT (b) shows two areas of focal uptake corresponding to fractures on axial SPECT/CT (c) in the anterior third (c, up) and fourth ribs (c, down). The patient was classified as “not metastatic.”

**Figure 2:**
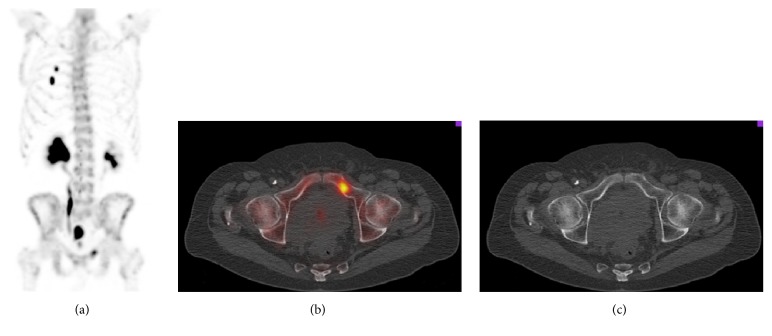
Whole-body SPECT/CT of the same patient as in Figure 1. Not only did the MIP-SPECT (a) show the 2 rib fractures seen in Figure 1, but also it showed an area of focal uptake in the superior pubic ramus. SPECT/CT fusion (b) shows focal uptake within the medullary bone without any correlative abnormality on the CT (c), and the patient was classified as “metastatic.”

It is well established that SPECT has better accuracy than planar scintigraphy and that SPECT/CT has better accuracy than SPECT alone [2–6, 10]. SPECT/CT performance can be explained by the physical properties of SPECT, which increase contrast and permit accurate localization in three dimensions, but also by the addition of CT images, which can contribute anatomic information to SPECT findings to identify metastases. For these reasons, SPECT/CT performance depends on CT parameters and image quality. Low-dose CT protocols are appropriate for the diagnosis of lytic bone changes [11]. The sensitivity and specificity of SPECT/CT were very high in our study, which is concordant with previous studies reporting values of 97% and 94%, respectively, on a per patient analysis [12]. The use of SPECT/CT has been widespread in recent years, but there are no specific guidelines concerning optimal imaging protocols to adopt for specific clinical indications [13]. Some authors use SPECT/CT only to identify equivocal lesions based on planar scintigraphy, but others recommend systematic whole-body SPECT/CT [4, 12, 14]. Our study compared whole-body SPECT/CT versus targeted SPECT/CT and showed a higher sensitivity with the whole-body SPECT/CT protocol than with targeted SPECT/CT. Whole-body SPECT/CT drastically changed the staging in an affirmative way in some cases: among 167 patients rated as “not metastatic” on planar with targeted SPECT/CT, four became “metastatic” on whole-body SPECT/CT (Figures 1 and 2). In other four cases, whole-body SPECT/CT changed an affirmative diagnosis obtained on planar scintigraphy with target SPECT/CT: among 167 patients “not metastatic” on planar with targeted SPECT/CT, four became “equivocal” on whole-body SPECT/CT. Conversely, among 40 patients rated as “metastatic” on planar with targeted SPECT/CT, three became “not metastatic” according to whole-body SPECT/CT. Five patients considered as “not metastatic” by planar scintigraphy with targeted SPECT/CT were metastatic according to the gold standard. On these five patients, four were classified as “metastatic” and one as “equivocal” by whole-body SPECT/CT. In total, whole-body SPECT/CT changed the diagnosis in 12 patients out of 212 (5.7%).

**Figure 3:**
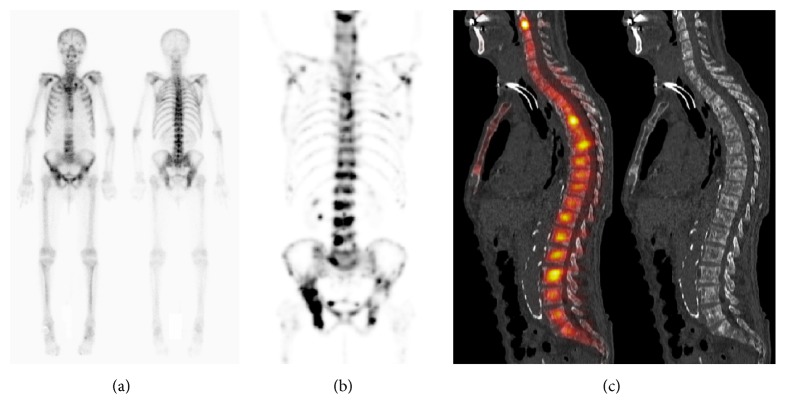
Prostate cancer patient with a history of carcinoma of the larynx. Markedly increased uptake relative to soft tissue, with absence of the urinary tract activity, consistent with Super Bone Scan on planar scintigraphy (a). MIP-SPECT (b) shows heterogeneous radiotracer uptake. Sagittal SPECT/CT (c) revealed diffuse metastases based on multiple areas of focal uptake and corresponding areas of sclerosis. The tracheotomy, in relationship with the treatment of the carcinoma of the larynx, is also visible on the images.

Bone superscan is a known pitfall and corresponds to diffuse bone infiltration of skeleton. It has been defined on planar scintigraphy by a markedly increased bone uptake relative to soft tissue, with absence or faint visualization of the urinary tract activity [15]. On SPECT/CT, all bone superscans were very easily identified as multimetastatic, because the higher contrast of SPECT increases heterogeneity of uptake, especially on MIP, and renal tracer elimination was not seen (Figure 3). This was often associated with an aspect of “moth eaten bone” on CT images. CT is thus very useful to confirm metastatic diagnosis and to differentiate it from metabolic bone diseases, such as renal osteodystrophy or secondary hyperparathyroidism due to renal failure, which can also occur in oncological patients [16].

**Figure 4:**
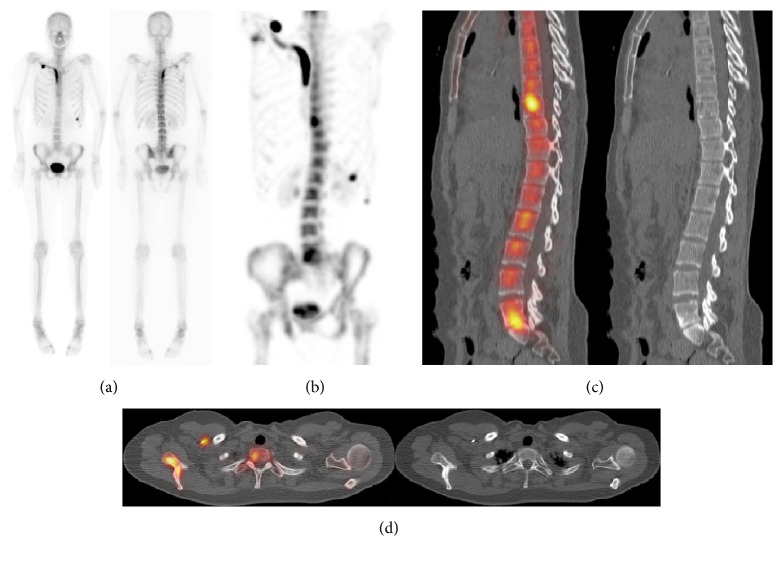
Breast cancer patient. Planar scintigraphy (a) showed artifactual uptake in a subclavian catheter, focal uptake at the 8th rib costochondral junction considered as a fracture, and heterogeneity of the thoracic spine without any focal uptake. MIP-SPECT (b) shows 3 additional areas of focal uptake: in L5-S1 corresponding to degenerative changes on the sagittal SPECT/CT (c), in the vertebral body of T9 (c), and in the right scapula (d) without lesion seen on CT, classified as “metastatic.”

Whole-body SPECT/CT has a higher sensitivity to identify extra-axial metastases, given the frequent involvement of the proximal portion of the limbs. Whole-body SPECT/CT was able to highlight extra-axial lesions in five patients (2.4%) not suspected on planar scintigraphy with targeted SPECT/CT (Figure 4). It is important to emphasize that the region with the higher risk of fracture on limbs, such as the femoral neck, can be carefully analyzed on SPECT/CT and was intentionally included within the field of view with this protocol.

The average CTDIvol (±SD) for a whole-body SPECT/CT was 3.76 ± 0.74 mGy and for one-field targeted SPECT/CT was half that at 1.88 mGy. The higher dose of whole-body SPECT/CT is worthwhile given its increased diagnostic sensitivity and potential to stage patients more accurately. Finally, a 22-minute whole-body SPECT/CT is not more time consuming than a planar scintigraphy (approximatively 13 minutes) with one additional targeted SPECT/CT acquisition (11 minutes by SPECT/CT field) and is less operator dependent.

One limitation of our study, inherent in its retrospective design, was the inhomogeneity of the reference standard including CT, MRI, PET, autopsy reports, and in some cases a subsequent scintigraphy ± SPECT/CT. Theoretically, the reference standard should be based on histology but that would have required a bone biopsy for every lesion, which is not practical or necessarily ethical. Although our data suggest that whole-body SPECT/CT is sufficient for oncologic workup, our population was inhomogeneous, including staging and restaging patients, with different types of cancers at different stage of diseases. Importantly, our results are based on a large population of patients with prostate and breast cancer, the two most frequent indications for bone scanning (overall 120 patients/212) (56.6%). It is necessary to validate these data with larger series for less common indications, such as lung and renal cancers, which might metastasize to the distal limbs before spreading to the axial skeleton [17, 18].

## 5. Conclusion

Whole-body SPECT/CT has a higher sensitivity than targeted SPECT/CT for detecting bone metastasis and changed the diagnosis in 12 patients out of 212 (5.7%) in our study. Moreover, this protocol increased detection rate of extra-axial lesions, particularly in the femoral neck, the area associated with the higher risk of pathologic fracture. In clinical practice, whole-body SPECT/CT alone, covering from the cervical region to the proximal femurs, should be the modality of choice, with an overall gain in camera time and reproducibility.

## Figures and Tables

**Table 1:** Repartition of scintigraphic findings on targeted SPECT/CT and whole-body SPECT/CT in relation to final diagnosis.

	*A*	*B*	*C*	Total
	Targeted SPECT/CT findings			
Final diagnosis				
Nonmetastatic	**162**	4	3	169
Equivocal	0	**0**	0	0
Metastatic	5	1	**37**	43

Total	167	5	40	212

	Whole-body SPECT/CT findings			
Final diagnosis				
Nonmetastatic	**163**	6	0	169
Equivocal	0	**0**	0	0
Metastatic	0	2	**41**	43

Total	163	8	41	212

*A*; nonmetastatic; *B* equivocal; *C* metastatic.

**Table 2:** Sensitivity and specificity 95% confidence intervals (CI) of planar with targeted SPECT/CT versus whole-body SPECT/CT.

	Threshold A		Threshold B	
	Sensitivity	Specificity	Sensitivity	Specificity
WB SPECT/CT	100 *(91.8; 100)*	96.4 *(92.4; 98.7)*	95.3 *(84.1; 99.4)*	100 *(97.8; 100)*
Targeted SPECT/CT	88.4 *(74.9; 96.1)*	95.9 *(91.7; 98.3)*	86.0 *(72.1; 94.7)*	98.2 *(94.9; 99.6)*
Difference and exact 95% CI	11.6 [1.9–25.1]	0.6 [−3.1–4.4]	9.3 [0.1–22.1]	1.8 [−0.6–5.1]
*p* value	0.0297	0.7949	0.0480	0.0977

**Table 3:** Repartition of extra-axial scintigraphic findings on targeted SPECT/CT and whole-body SPECT/CT in relation to final diagnosis.

	*A*	*B*	*C*	Total
	Targeted SPECT/CT findings			
Final diagnosis				
Nonmetastatic	**179**	4	2	185
Equivocal	0	**0**	0	0
Metastatic	6	0	**21**	27

Total	185	4	23	212

	Whole-body SPECT/CT findings			
Final diagnosis				
Nonmetastatic	**180**	3	2	185
Equivocal	0	**0**	0	0
Metastatic	1	0	**26**	27

Total	181	3	28	212

*A*; nonmetastatic; *B* equivocal; *C* metastatic.

**Table 4:** Sensitivity and specificity 95% confidence intervals (CI) of planar with targeted SPECT/CT versus whole-body SPECT/CT for extra-axial lesions.

	Threshold A		Threshold B	
	Sensitivity	Specificity	Sensitivity	Specificity
WB SPECT/CT	96.3 *(81.0; 99.9)*	97.3 *(93.8; 99.1)*	96.3 *(81.0; 99.9)*	98.9 *(96.1; 99.9)*
Targeted SPECT/CT	77.8 *(57.7; 91.4)*	96.8 *(93.1; 98.8)*	77.8 *(57.7; 91.4)*	98.9 *(96.1; 99.9)*
Difference and exact 95% CI	18.5 [2.9–38.1]	0.5 [−1.5–3.0]	18.5 [2.9–38.1]	No discordant pairs
*p* value	0.0266	0.5279	0.0266	1
